# Matrix-Based Formulation of Heterogeneous Individual-Based Models of Infectious Diseases: Using SARS Epidemic as a Case Study

**DOI:** 10.3390/ijerph18115716

**Published:** 2021-05-26

**Authors:** Wei Duan

**Affiliations:** College of Systems Engineering, National University of Defense Technology, Changsha 410073, China; duanwei@nudt.edu.cn

**Keywords:** public health, epidemic modeling, agent-based models, heterogeneity, algorithms

## Abstract

Heterogeneities of individual attributes and behaviors play an important role in the complex process of epidemic spreading. Compared to differential equation-based system dynamical models of infectious disease transmission, individual-based epidemic models exhibit the advantage of providing a more detailed description of realities to capture heterogeneities across a population. However, the higher granularity and resolution of individual-based epidemic models comes with the cost of increased computational complexities, which result in difficulty in formulating individual-based epidemic models with mathematics. Furthermore, it requires great effort to understand and reproduce existing individual-based epidemic models presented by previous researchers. We proposed a mathematical formulation of heterogeneous individual-based epidemic models using matrices. Matrices and vectors were applied to represent individual attributes and behaviors. We derived analytical results from the matrix-based formulations of individual epidemic models, and then designed algorithms to force the computation of matrix-based individual epidemic models. Finally, we used a SARS epidemic control as a case study to verify the matrix-based formulation of heterogeneous individual-based epidemic models.

## 1. Introduction

Heterogeneity among the units composing a system is a very generic feature, which means that one never finds two units behaving in exactly the same way in most real systems [1]. In human societies, individual variability widely exists in social settings, cultural backgrounds, ages, genders, and occupations. Human beings have diverse features, physical characteristics, activities, and contact patterns. In epidemic outbreaks, individuals exhibit various health states, infectiousness, epidemic progresses, and behavior response. Despite these general facts, it is usually assumed that a population is homogeneous so as to build analytically tractable epidemic models, such as differential equation-based system dynamical models [2,3].

However, recent studies have shown that individual heterogeneity plays an important role in the epidemic spreading process [4,5,6]. The impact that individual heterogeneity has on epidemic diffusion has increasingly received more attention. Some researchers have concentrated on the effect of heterogeneous populations on epidemic dynamics [6,7,8]. They incorporated the heterogeneities of host populations as state variables, including age, stage, size [8], infection threshold [7], and resistant hosts [6]. Some other researchers have focused on epidemic models with spatial heterogeneities [9,10,11]. They utilized a patch structure where an epidemic system was divided into a number of patches to represent different spatial characteristics. Many researchers also investigated the impact of individuals’ heterogeneous behaviors and activities on epidemic diffusion [12,13,14,15]. Yang et al. [12] studied the impact of heterogeneous human activities on epidemic spread and found that the heterogeneity of activities affects spreading velocity remarkably. Merler et al. also studied how human motilities and population heterogeneity affect the course of an epidemic [13]. They found that the cumulative attack rate is positively correlated with average household size and the fraction of students in a population, and this was negatively correlated with the fraction of inactive population. Shang addressed the impact of awareness on epidemic outbreaks by proposing a mean-filed approach accommodating heterogeneous transmission rates [14]. He found that both contact awareness and local awareness can raise the epidemic threshold, while global awareness cannot. Cui et al. applied an edge-based compartmental model to study epidemic spreading dynamics with heterogeneous contacts [15]. In their models, contacts were grouped into two classes: strong contacts and normal ones. Additionally, the super spreading events that emerged in the severe acute respiratory syndrome (SARS) epidemic outbreaks of 2003 highlighted the necessity of a better understanding of social heterogeneities and individual-based models [16]. When studying super spreading events of the SARS epidemic using differential equation models, Mkhatshwa et al. [17] divided infectious individuals into two subgroups: super spreaders and regular spreaders. They then assigned a higher infection rate to super spreaders. Duan et al. employed heterogeneous and stochastic agent-based models to analyze the characteristics of infectious diseases’ super spreaders [18]. Researchers also paid attention to epidemic dynamics in heterogeneous networks with different connectivities [19,20,21], and went forward and investigated epidemic spread in weighted networks representing the heterogeneity of individual interaction strengths [22,23,24,25].

Differential equation models and individual-based models are the two most widely used approaches of representing epidemic diffusion [26]. The former makes some reasonable assumptions and simplifications so as to represent epidemic spread at a macro level [2]. A population is assumed to be homogeneous, well-mixed, and aggregated into several compartments according to people’s health states. The transitions of population between compartments are described by using differential equations with variables, such as the infection rate, onset rate of symptoms, and recovery rate. These assumptions and simplifications endow differential equation models with the advantage of performing theoretical analysis of macroscopic regularities of epidemic spread, such as the epidemic threshold and the final epidemic size. However, the advantage also comes with the limitation in representing epidemic diffusion in detail [27]. Firstly, the assumption of homogeneous and well-mixed population results in difficulties in representing the variants of individual microscopic attributes and behaviors. Secondly, a small set of variables parameterized with average quantities and mean values are inadequate to capture a variety of factors associated with the epidemic spreading process.

The individual-based model is a promising modeling paradigm to represent individual heterogeneities [28,29]. This approach can provide a more detailed depiction of realities to capture heterogeneity across individuals and incorporate the stochastic nature of infectious disease transmission. However, the higher granularity and resolution provided by individual-based models comes with the cost of increased computational complexities, growing computational power, and the development of intelligent computational algorithms [30]. The complexity of heterogeneous individual-based models leads to difficulty in mathematical formulation. Sometimes, heterogeneous individual-based epidemic models do not provide an insight into technical details, but only some qualitative representations. Consequently, we need to make great efforts to understand and reproduce existing individual-based epidemic models.

Our purpose is to realize the mathematical formulation of heterogeneous individual-based epidemic models, as well as make individual-based epidemic models much easier to be understood and reproduced. We used matrices and vectors to represent individuals, activity locations, social organizations, and relationship networks. Matrix-based representation of individual-based epidemic models reserves the capability of describing the heterogeneity of individuals and epidemic spreading process [8]. Furthermore, all of factors or elements related to epidemic diffusion are described as specific variables in matrices or vectors. According to the specific variables, it is feasible to build a mathematical formulation of individual-based epidemic models and algorithms. In addition, we can derive analytical results from the matrix-based representation of individual-based epidemic models. We applied the SARS epidemic as a case study to verify the matrix-based formulation of heterogeneous individual-based epidemic models.

## 2. Heterogeneous Individual-Based Models of SARS Epidemic

We here presented an improved agent-based model of the SARS epidemic based on our previous works, found in reference [18], which were used as a case study of matrix-based formulations of heterogeneous individual-based epidemic models. The main improvement of the model here is to increase the presentation of individuals’ daily commute activities among spatial locations into our previous works so as to study the heterogeneous spatial transmission pattern of the SARS epidemic. Consequently, we designed individuals’ daily activities at spatial locations, and introduced a bipartite network to describe individuals’ commute behaviors. We assumed a closed human society to study the spread and control of the SARS epidemic. We applied agent-based models to represent human individuals, spatial activity locations, social organizations in the closed society, and their behaviors. In addition, we employed complex networks to described the multi-relationships among these social entities. We designed individuals’ daily activities, such as sleeping, working, shopping, and recreation, which take place at different spatial activity locations. Agents commute among spatial locations to execute daily activities and contact each other. The contacts between susceptible individuals and infectious individuals cause the transmission of the SARS pathogen. Social organizations take some non-pharmaceutical interventions to mitigate the transmission of SARS epidemic, such as contact tracing, spatial activity location closure, individual quarantine, which may change the states of individuals and spatial activity locations. We then computed the discrete events and updated the states of social entities in time sequence to simulate the spread and control of the SARS epidemic.

### 2.1. Individuals’ Daily Commute Patterns

We represented individuals’ daily activities as mobility events, and randomly scheduled these discrete events in queuing models. We assumed individuals executed $k^{m}$ mobility activity events in each day.

We used a bipartite network with time-varying weights to model individuals’ daily commute patterns. There are two types of vertices in the bipartite network, including individual vertices and spatial location vertices. Network edges only exist between individual vertices and spatial location vertices. Edge weights denote the propensity that individuals visit spatial locations.

The bipartite network was designed in term of individuals’ living regularities. On account of the power-law characteristics of human dynamics [31], the edge weights of bipartite network were generated by a truncated power-law random variable described as

(1) $$w\left(r\right)={\left(w_{\min}^{-\rho +1}+\left(w_{\max}^{-\rho +1}-w_{\min}^{-\rho +1}\right)r\right)}^{\frac{1}{-\rho +1}},$$

where $w\left(r\right)\in \left[w_{\min},w_{\max}\right]$ is a truncated power-law random variable. r∈[0,1] is a uniform random number. ρ is the exponent of the power-law distribution. The probability that an individual moves to a spatial location in a commute activity is defined as

(2) $$p_{ij}\left(t\right)=\frac{w_{ij}\left(t\right)}{\sum_{n_{i}\left(t\right)}w_{ik}\left(t\right)},$$

where $p_{ij}\left(t\right)$ is the probability that individual *i* move to spatial activity location *j*. $w_{ij}\left(t\right)$ is the edge weight between individual *i* and spatial location *j* at time *t*. $n_{i}\left(t\right)$ is the number of spatial locations individual *i* could visit at time *t*. It means spatial locations closed in the SARS epidemic outbreak is not allowed to be visited by individual *i* at time *t*.

### 2.2. Individuals’ Structured Contact Patterns

We also represented individuals’ contact activities as contact events, and randomly scheduled contact events in queuing models. According to survey results of human daily contacts [32], we used a normal random variable to represent the number of individuals’ daily contacts, which is described as

(3) $$k^{c}\left(r_{1},r_{2}\right)=\mu +\sigma {\left(-2\ln r_{1}\right)}^{\frac{1}{2}}\cos\left(2\pi r_{2}\right),$$

where $k^{c}$ is the random variable of individuals’ daily contact number. $r_{1}$ and $r_{2}$ are both uniform random numbers in the range [0, 1]. In addition, considering the statistical features of human behaviors, we designed the time duration of each contact activity as a power-law waiting time distribution described as

(4) $$d\left(r\right)={\left(d_{\min}^{-\rho +1}+\left(d_{\max}^{-\rho +1}-d_{\min}^{-\rho +1}\right)r\right)}^{\frac{1}{-\rho +1}},$$

where d(r) is a random variable denoting the time duration of individuals’ contacts. r∈[0,1] is a uniform random number. ρ is the exponent of the power-law distribution.

We built a contact network in term of individuals’ social relationships to represent individual contact patterns. The edge weights of contact network were generated by the formulation described as

(5) $$w_{ij}=w_{0}{\left(n_{i}n_{i}\right)}^{\theta },$$

where $w_{ij}$ is the edge weight between individual *i* and individual *j*. $w_{0}$ is the proportional parameter. $n_{i}$ and $n_{j}$ are the node degrees of individual *i* and individual *j*, respectively. Once individual *i* initiates a contact activity, it selects a neighbor in its personal contact network as a contact object according to the following rule

(6) $$q_{ij}\left(t\right)=\frac{w_{ij}\left(t\right)}{\sum_{n_{i}\left(t\right)}w_{ik}\left(t\right)},$$

where $q_{ij}\left(t\right)$ is the probability that individual *i* selects individual *j* as a contact object. $w_{ij}\left(t\right)$ here is the edge weight between individual *i* and individual *j* in the contact network. $n_{i}\left(t\right)$ here is the number of neighbors in personal contact networks of individual *i*, which could be contacted by individual *i*. It means individuals who are quarantined or are not at the same spatial activity location cannot be contacted by individual *i*.

### 2.3. Infection Probability of SARS

SARS pathogens are transmitted from infectious individuals to susceptible individuals in their face-to-face contacts. Infectious individuals shed SARS pathogens through aerosols and droplets to susceptible individuals. Once susceptible individuals inhale a sufficient dose of SARS pathogens, they are infected by the SARS pathogen. Consequently, the infection probability of the SARS epidemic in a single contact is related to contact intensity and duration, infectivity of infectious individuals, and immunity of susceptible individuals. Here, we only considerer contact duration and infectivity. We assumed the infection probability of SARS epidemic in a single contact is

(7) Pro=Dur×In,

where Dur is the time duration of a single contact between a susceptible individual and an infectious individual. In is the infectivity of the infectious individual. The infectivity is related to pathogen load and shedding rates of infectious individuals, and evolves along with the infectious period. According to consensus documents [33], we used a triangle distribution to model the infectivity (*Inf*) of infectious individuals, which is described as

(8) $$f^{Inf}\left(t\right)=\{\begin{array}{c} \frac{2\left(t-t_{0}\right)}{\left(t^{*}-t_{0}\right)\left(t_{1}-t_{0}\right)},t_{0}\leq t\leq t^{*}\\ \frac{2\left(t_{1}-t\right)}{\left(t_{1}-t^{*}\right)\left(t_{1}-t_{0}\right)},t^{*}<t\leq t_{1} \end{array},$$

where $t_{0}$ and $t_{1}$ are the time points of beginning and ending infectious period. $t^{*}$ is the time point when infectious individuals have the max infectivity, which is set as $t^{*}=t_{0}+10$ day. According to Reference [18], we fitted the infection probability (*Inf-Pro*) of SARS in case infectious individuals have the max infectivity as

(9) $$f^{Inf-Pro}\left(d\right)=\left(1-e^{-0.019d}\right),t_{0}\leq t\leq t_{1},$$

where d is the time duration of a contact between an infectious individual and a susceptible individual. We then designed infection probability by the proportion of infectivity with the max infectivity as

(10) $$f^{Inf-Pro}\left(t,d\right)=\{\begin{array}{c} \left(1-e^{-0.019d}\right)\frac{t-t_{0}}{t^{*}-t_{0}},t_{0}\leq t\leq t^{*}\\ \left(1-e^{-0.019d}\right)\frac{t_{1}-t}{t_{1}-t^{*}},t^{*}<t\leq t_{1} \end{array},$$

where t is the time of infectious period.

### 2.4. Heterogeneous Epidemic Progress of SARS

We assumed each infected individual went through four health state period, including susceptible period, latent period, infectious period, and recovered period. A susceptible individual is infected by the SARS epidemic in a single contact with an infectious individual. Then, the health state of the susceptible individual become latent. Several days later, the individual goes over the latent period, and become in infectious state. The infectious individual exhibits the capability of transmitting SARS pathogens to other susceptible individuals. On account of the onset of symptoms, infectious individuals will be admitted as patient cases of the SARS epidemic in hospitals. Admitted cases receives treatment in hospitals, and are quarantined until they recover from infectious state. Recovered individuals could be discharged from hospitals as they will not be infected by SARS epidemic again.

The duration of health state periods, admission time, and discharge time are fitted as Gamma distributions, of which the means (standard deviation) were 6.37 (16.69), 23.5 (62.1), 4.85 (12.19), and 23.1 (62.1) days, respectively [34]. To build the heterogeneous presentation of SARS progress, we applied Gamma random variables to scale the SARS epidemic progress.

## 3. Matrix-Based Formulation of the Models

To make heterogeneous individual-based models of SARS epidemic above much easier to be understood and reproduced, we applied matrices and vectors to describe objects and entities in the closed society. More technical details related to models and algorithms are represented in the matrix-based formulation. We firstly defined the models’ framework as a group of sets formulated as {M(t)*,* C(t)*,* S(t)*,* $A\left(a_{k}\right)$}. M(t) is the set of matrices representing entities and their attributes, such as individuals, spatial locations, health states. C(t) is the set of constraint conditions for entity matrices set M(t). S(t) is the set of analytical results derived from M(t). $A\left(a_{k}\right)$ is the set of algorithms advancing the computation of heterogeneous individual-based epidemic models formulated by matrices.

### 3.1. Matrix-Based Formulation of Entities

#### 3.1.1. Formulation of Individuals and Their Attributes

We assumed a closed population consisted of $N^{Ind}$ ($N^{Ind}\in Z^{+}$) individuals (*Ind*) in epidemic system. Individuals were represented as

(11) $$M^{Ind}={\left[\begin{array}{cccc} a_{1}^{Ind} & a_{2}^{Ind} & \cdots & a_{n}^{Ind} \end{array}\right]}^{T},$$

where $M^{Ind}$ is individual matrix with *n* rows and 1 column. $a_{i}^{Ind}$ is individual *i* in the population. The constraint condition of individual matrix $M^{Ind}$ is defined as $C^{Ind}=\left\{n=N^{Ind}\right\}$, which means the row of individual matrix $M^{Ind}$ equals to the number of individuals in the population. Similarly, we used matrices to formulate individuals’ attributes, such as health states, epidemic progresses, infectiousness, and immunity.

We formulated individuals’ health states (*HS*) as

(12) $$M^{HS}\left(t\right)=\left[\begin{array}{ccc} a_{11\left(x\right)}^{HS}\left(t\right) & \cdots & a_{1h\left(x\right)}^{HS}\left(t\right)\\ \vdots & \ddots & \vdots \\ a_{n1\left(x\right)}^{HS}\left(t\right) & \cdots & a_{nh\left(x\right)}^{HS}\left(t\right) \end{array}\right],$$

where $M^{HS}\left(t\right)$ is health state matrix with *n* rows and *h* columns. *h* is the number of individual health states in epidemic progresses. $a_{ij\left(x\right)}^{HS}\left(t\right)=1$ means individual $a_{i}^{Ind}$ is in health state *j* (*x*) at time *t*. Susceptible state (*S*), latent state (*L*), infectious state (*I*), and recovered state (*R*) are marked as x∈{S,L,I,R}, respectively. The constraint conditions $C^{HS}\left(t\right)$ of health state matrix $M^{HS}\left(t\right)$ include $n=N^{Ind}$, $a_{ij\left(x\right)}^{HS}\left(t\right)\in \left\{0,1\right\}$ denoting the label of health state *j* of individual $a_{i}^{Ind}$ at time *t*, $\sum_{j}a_{ij\left(x\right)}^{HS}\left(t\right)=1$ meaning individual $a_{i}^{Ind}$ have to be in only one health state at time *t*, and $\sum_{i}\sum_{j}a_{ij\left(x\right)}^{HS}\left(t\right)=N^{Ind}$ denoting all individuals are in one of health states at time *t*.

Epidemic progresses describe the transitions of individuals among different health periods. Individuals go through different periods of health states, which occupy different time durations, such as latent period, infectious period, and recovered period. We described epidemic progresses (*EP*) based on heterogeneous time scales as

(13) $$M^{EP}=\left[\begin{array}{ccc} a_{11\left(x\right)}^{EP} & \cdots & a_{1m\left(x\right)}^{EP}\\ \vdots & \ddots & \vdots \\ a_{n1\left(x\right)}^{EP} & \cdots & a_{nm\left(x\right)}^{EP} \end{array}\right],$$

where $M^{EP}$ is epidemic progress matrix with *n* rows and *m* columns. $a_{ij\left(x\right)}^{EP}$ is the time duration of individual $a_{i}^{Ind}$ in the period of health state *j* (*x*). We described infection time (*IT*), latent period (*LP*), infectious period (*IP*), admission time (*AT*), and discharge time (*DT*) of individual $a_{i}^{Ind}$ as $a_{i1\left(IT\right)}^{EP}$, $a_{i2\left(LP\right)}^{EP}$, $a_{i3\left(IP\right)}^{EP}$, $a_{i4\left(AT\right)}^{EP}$, and $a_{i5\left(DT\right)}^{EP}$, respectively. The constraint conditions $C^{EP}$ of epidemic progress matrix $M^{EP}$ include $n=N^{Ind}$, $a_{ij\left(x\right)}^{EP}=f^{EP}\left(x,t\right)$ meaning the time duration of health state period comes from a certain function, which is a stochastic Gamma distribution or a constant value, and $a_{i1\left(S\right)}^{HS}=1\Rightarrow a_{ij\left(S\right)}^{EP}=0$ meaning in case individual $a_{i}^{Ind}$ is not infected, all time durations of health state period are 0.

We applied a vector to represent the infectivity (*Inf*) of infectious individuals as

(14) $$M^{Inf}\left(t\right)={\left[\begin{array}{cccc} a_{1}^{Inf}\left(t\right) & a_{2}^{Inf}\left(t\right) & \cdots & a_{n}^{Inf}\left(t\right) \end{array}\right]}^{T},$$

where $M^{Inf}\left(t\right)$ is infectivity matrix with *n* rows and 1 column. $a_{i}^{Inf}\left(t\right)$ is the infectivity of individual $a_{i}^{Ind}$ at time *t*. The constraint conditions $C^{Inf}\left(t\right)$ of infectivity matrix $M^{Inf}\left(t\right)$ include $n=N^{Ind}$, $a_{i}^{Inf}\left(t\right)=f^{Inf}\left(t\right)$ meaning the infectivity of infectious individuals is a function defined in Equation (8), and $a_{i3\left(I\right)}^{HS}=0\Rightarrow a_{i}^{Inf}\left(t\right)=0$ denoting individuals have infectivity only in case they are in infectious state.

Recovered individuals exhibit diverse abilities to resist pathogen. We formulated the immunity (*Imm*) of individuals as

(15) $$M^{Imm}\left(t\right)={\left[\begin{array}{cccc} a_{1}^{Imm}\left(t\right) & a_{2}^{Imm}\left(t\right) & \cdots & a_{n}^{Imm}\left(t\right) \end{array}\right]}^{T},$$

where $M^{Imm}\left(t\right)$ is immunity matrix with *n* rows and 1 column. $a_{i}^{Imm}\left(t\right)$ is the immunity of individual $a_{i}^{Ind}$ at time *t*. The constraint conditions $C^{Imm}\left(t\right)$ of immunity matrix $M^{Imm}\left(t\right)$ include $n=N^{Ind}$, $a_{i}^{Imm}\left(t\right)=f^{Imm}\left(t\right)$ meaning the immunity of individuals is a certain function, and $a_{i4\left(R\right)}^{HS}=0\Rightarrow a_{i}^{Imm}\left(t\right)=0$ denoting individuals exhibit immunity in case they are in recovered state.

Once individuals are admitted as patients in hospitals, they are quarantined and prohibited to move outside and contact with others. We formulated individuals’ quarantine (*Qua*) state as

(16) $$M^{\mathrm{Qua}}\left(t\right)={\left[\begin{array}{cccc} a_{1}^{Qua}\left(t\right) & a_{2}^{Qua}\left(t\right) & \cdots & a_{n}^{Qua}\left(t\right) \end{array}\right]}^{T},$$

where $M^{Qua}\left(t\right)$ is quarantine matrix with *n* rows. $a_{i}^{Qua}\left(t\right)$ is label denoting whether individual $a_{i}^{Ind}$ is quarantined. The constraint conditions $C^{Qua}\left(t\right)$ of quarantine matrix include $n=N^{Ind}$ and $a_{i}^{Qua}\left(t\right)\in \left\{0,1\right\}$.

#### 3.1.2. Formulation of Spatial Locations and Their Attributes

Human mobility patterns depend on the transmission routes of infectious diseases. We assumed that a closed environment consists of many spatial locations. The number of spatial locations (*SL*) was assumed as $N^{\mathrm{SL}}\in Z^{+}$. The spatial location matrix is formulated as

(17) $$M^{SL}={\left[\begin{array}{cccc} a_{1}^{SL} & a_{2}^{SL} & \cdots & a_{l}^{SL} \end{array}\right]}^{T},$$

where $a_{i}^{SL}$ is spatial location *i* in the scene. The constraint condition $C^{SL}$ of spatial location matrix $M^{\mathrm{SL}}$ is $l=N^{SL}$, which means the number of rows of spatial location matrix equals to the number of spatial locations in the closed environment.

During epidemic outbreaks, non-pharmaceutical interventions may be employed to close some spatial locations, such as campus, restaurants, and cinemas. We formulated the closure (*Clo*) state of spatial locations as

(18) $$M^{Clo}\left(t\right)={\left[\begin{array}{cccc} a_{1}^{Clo}\left(t\right) & a_{2}^{Clo}\left(t\right) & \cdots & a_{l}^{Clo}\left(t\right) \end{array}\right]}^{T},$$

where $M^{Clo}\left(t\right)$ is closure state matrix with *l* rows. The constraint conditions $C^{Clo}\left(t\right)$ of closure state matrix $M^{Clo}\left(t\right)$ include $l=N^{SL}$ and $a_{i}^{Clo}\left(t\right)\in \left\{0,1\right\}$.

#### 3.1.3. Formulation of Social Organizations and Their Attributes

We assumed there were $N^{\mathrm{Org}}\in Z^{+}$ social organizations (*Org*) in the closed environment, and formulated social organizations as

(19) $$M^{Org}={\left[\begin{array}{cccc} a_{1}^{Org} & a_{2}^{Org} & \cdots & a_{g}^{Org} \end{array}\right]}^{T},$$

where $M^{Org}$ is social organization matrix with *g* rows. $a_{i}^{Org}$ is social organization *i*. The constraint condition $C^{Org}$ of social organization matrix is $g=N^{Org}$, which means the rows of social organization matrix equals to the number of social organizations.

Considering social organizations own spatial locations, such as office buildings, we formulated the ownerships between social organizations and spatial locations (*Org-SL*) as

(20) $$M^{Org-SL}=\left[\begin{array}{ccc} a_{11}^{Org-SL} & \cdots & a_{1l}^{Org-SL}\\ \vdots & \ddots & \vdots \\ a_{g1}^{Org-SL} & \cdots & a_{gl}^{Org-SL} \end{array}\right],$$

where $M^{Org-SL}$ is called as organization-location relationship matrix with *g* rows and *l* columns. $a_{ij}^{Org-SL}$ is the label denoting whether social organization $a_{i}^{Org}$ is sited in spatial location $a_{j}^{SL}$. The constraint conditions $C^{Org-SL}$ of organization-location relationship matrix including $g=N^{Org}$, $l=N^{SL}$, and $a_{ij}^{Org-SL}\in \left\{0,1\right\}$. Furthermore, we also formulated the relationship between social organizations and individuals (*Org-Ind*) as

(21) $$M^{Org-Ind}=\left[\begin{array}{ccc} a_{11}^{Org-Ind} & \cdots & a_{1g}^{Org-Ind}\\ \vdots & \ddots & \vdots \\ a_{n1}^{Org-Ind} & \cdots & a_{ng}^{Org-Ind} \end{array}\right],$$

where $M^{Org-Ind}$ is organization-individual relationship matrix with *n* rows and *g* columns. $a_{ij}^{Org-Ind}$ is the label denoting whether individual $a_{i}^{Ind}$ belongs to social organization $a_{j}^{Org}$. The constraint conditions $C^{Org-Ind}$ of organization-individual relationship matrix include $n=N^{Ind}$, $g=N^{Org}$, and $a_{ij}^{Org-Ind}\in \left\{0,1\right\}$.

#### 3.1.4. Formulation of Weighted Bipartite Networks

We applied a weighted bipartite network to represent individuals’ commute patterns among spatial locations. There are two types of vertices in the bipartite network, which denote individuals and spatial locations, respectively. Network edges only exist between individuals and spatial locations. Edge weights represent the propensity that individuals visit spatial locations. We formulated the bipartite network (*BN*) as

(22) $$M^{BN}=\left[\begin{array}{ccc} a_{11}^{BN} & \cdots & a_{1l}^{BN}\\ \vdots & \ddots & \vdots \\ a_{n1}^{BN} & \cdots & a_{nl}^{BN} \end{array}\right],$$

where $M^{BN}$ is bipartite network matrix with *n* rows and *l* columns. $a_{ij}^{BN}$ is the label denoting whether individual $a_{i}^{Ind}$ is linked to spatial location $a_{j}^{SL}$. The constraint conditions $C^{BN}$ of bipartite network matrix include $n=N^{Ind}$, $l=N^{SL}$, and $a_{ij}^{BN}\in \left\{0,1\right\}$. The edge weights of bipartite network (*BNW*) are formulated as

(23) $$M^{BNW}\left(t\right)=\left[\begin{array}{ccc} a_{11}^{BNW}\left(t\right) & \cdots & a_{1l}^{BNW}\left(t\right)\\ \vdots & \ddots & \vdots \\ a_{n1}^{BNW}\left(t\right) & \cdots & a_{nl}^{BNW}\left(t\right) \end{array}\right],$$

where $M^{BNW}\left(t\right)$ is the edge weights matrix of bipartite network with *n* rows and *l* columns. $a_{ij}^{BNW}\left(t\right)$ is the edge weight between individual $a_{i}^{Ind}$ and spatial location $a_{j}^{SL}$ at time *t*. The constraint conditions $C^{BNW}\left(t\right)$ of edge weights matrix of bipartite network include $n=N^{Ind}$, $l=N^{SL}$, $a_{ij}^{BNW}\left(t\right)=f^{BNW}\left(t,i,j\right)⇐\omega \left(r\right)$ meaning the edge weights of bipartite network are generated by a function defined in Equation (1), and $a_{ij}^{BN}=0\Rightarrow a_{ij}^{BNW}\left(t\right)=0$ meaning the edge weight is 0 in case individual $a_{i}^{Ind}$ is not connected to spatial location $a_{j}^{SL}$.

Individuals’ current spatial positions (*CSL*) at time *t* are recorded as

(24) $$M^{CSL}\left(t\right)=\left[\begin{array}{ccc} a_{11}^{CSL}\left(t\right) & \cdots & a_{1l}^{CSL}\left(t\right)\\ \vdots & \ddots & \vdots \\ a_{n1}^{CSL}\left(t\right) & \cdots & a_{nl}^{CSL}\left(t\right) \end{array}\right],$$

where $M^{CSL}\left(t\right)$ is individual current spatial location matrix with *n* rows and *m* columns. $a_{ij}^{CSL}\left(t\right)$ is the label denoting whether individual $a_{i}^{Ind}$ is at spatial location $a_{j}^{SL}$ at time *t*. The constraint conditions $C^{CSL}\left(t\right)$ of individual current spatial location matrix include $n=N^{Ind}$, $l=N^{SL}$, $a_{ij}^{CSL}\left(t\right)\in \left\{0,1\right\}$, $\sum_{j}a_{ij}^{CSL}\left(t\right)=1$ meaning individual $a_{i}^{Ind}$ have to stay at only one spatial location, and $\sum_{i}\sum_{j}a_{ij}^{CSL}\left(t\right)=N^{Ind}$ meaning all individuals stay at the spatial locations.

#### 3.1.5. Formulation of Weighted Contact Networks

We represented individuals’ heterogeneous contact patterns by using a weighted contact network, where nodes stood for individuals, edges represented individuals’ relationships, and edge weights described individuals’ interaction strength. We formulated the contact network (*CN*) as

(25) $$M^{CN}=\left[\begin{array}{ccc} a_{11}^{CN} & \cdots & a_{1n}^{CN}\\ \vdots & \ddots & \vdots \\ a_{n1}^{CN} & \cdots & a_{nn}^{CN} \end{array}\right],$$

where $M^{CN}$ is contact network matrix with *n* rows and *n* columns. $a_{ij}^{CN}$ is the label denoting whether individual $a_{i}^{Ind}$ is connected to individual $a_{j}^{Ind}$. The constraint conditions $C^{CN}$ of contact network matrix include $n=N^{Ind}$, $a_{ii}^{CN}=0$, and $a_{ij}^{CN}\in \left\{0,1\right\}$. Edge weights of the contact network (*CNW*) were formulated as

(26) $$M^{CNW}\left(t\right)=\left[\begin{array}{ccc} a_{11}^{CNW}\left(t\right) & \cdots & a_{1n}^{CNW}\left(t\right)\\ \vdots & \ddots & \vdots \\ a_{n1}^{CNW}\left(t\right) & \cdots & a_{nn}^{CNW}\left(t\right) \end{array}\right],$$

where $M^{CNW}\left(t\right)$ is the edge weight matrix of contact network with *n* rows and *n* columns. $a_{ij}^{CNW}\left(t\right)$ is the edge weight between individual $a_{i}^{Ind}$ and individual $a_{j}^{Ind}$ at time *t*. The constraint conditions $C^{CNW}$ of edge weight matrix of contact network include $n=N^{Ind}$, $a_{ij}^{CNW}\left(t\right)=f^{CNW}\left(t,i,j\right)⇐\omega_{ij}$ meaning the edge weights of contact network is generated by a certain function defined in Equation (5), and $a_{ij}^{CN}=0\Rightarrow a_{ij}^{CNW}\left(t\right)=0$ meaning the edge weight between two individuals is 0 in case they are not connected with each other. We marked who were contacting with whom as

(27) $$M^{CO}\left(t\right)=\left[\begin{array}{ccc} a_{11}^{CO}\left(t\right) & \cdots & a_{1n}^{CO}\left(t\right)\\ \vdots & \ddots & \vdots \\ a_{n1}^{CO}\left(t\right) & \cdots & a_{nn}^{CO}\left(t\right) \end{array}\right],$$

where $M^{CO}\left(t\right)$ is contact object (*CO*) matrix. $a_{ij}^{CO}\left(t\right)$ is the label denoting whether individual $a_{i}^{Ind}$ is contacting with individual $a_{j}^{Ind}$ at time *t*. The constraint conditions $C^{CO}$ of contact object matrix include $n=N^{Ind}$, $a_{ij}^{CO}\left(t\right)\in \left\{0,1\right\}$, and $a_{ii}^{CO}\left(t\right)=0$ meaning individuals cannot contact with themselves.

#### 3.1.6. Formulation of Transmission Networks

We recorded the transmission networks of epidemics to analyze transmission routes of epidemics and derive the reproduce number of infected cases. The epidemic transmission network (*TN*) is represented as

(28) $$M^{TN}=\left[\begin{array}{ccc} a_{11}^{TN} & \cdots & a_{1n}^{TN}\\ \vdots & \ddots & \vdots \\ a_{n1}^{TN} & \cdots & a_{nn}^{TN} \end{array}\right],$$

where $a_{ij}^{TN}$ is the label denoting whether individual $a_{i}^{Ind}$ is infected by epidemic from individual $a_{j}^{Ind}$. The constraint conditions $C^{TN}$ of transmission network matrix include $n=N^{Ind}$, $a_{ij}^{TN}\in \left\{0,1\right\}$, and $a_{ii}^{TN}=0$ meaning individuals cannot transmit epidemics to themselves.

#### 3.1.7. Formulation of Individuals’ Mobility Activities

We represented the heterogeneous mobility patterns of individuals, and scheduled individuals’ mobility activities as discrete events in queue models. Individual mobility event (*ME*) matrix is formulated as

(29) $$M^{ME}=\left[\begin{array}{ccc} a_{11}^{ME}\left(t_{1},l_{1}\right) & \cdots & a_{1k^{m}}^{ME}\left(t_{k^{m}},l_{k^{m}}\right)\\ \vdots & \ddots & \vdots \\ a_{n1}^{ME}\left(t_{1},l_{1}\right) & \cdots & a_{nk^{m}}^{ME}\left(t_{k^{m}},l_{k^{m}}\right) \end{array}\right],$$

where $M^{ME}$ is mobility event matrix with *n* rows and *k^m^* columns. $a_{ij}^{ME}\left(t_{j},l_{j}\right)$ is the mobility event *j* of individual $a_{i}^{Ind}$, which denotes individual $a_{i}^{Ind}$ will move to spatial location $l_{j}\in M^{SL}$ at time $t_{j}$. The constraint conditions $C^{ME}$ of mobility event matrix include $i<j\Rightarrow t_{i}<t_{j}$ meaning mobility events are scheduled in time sequence, $t_{j}=f^{METP}\left(t\right)$ meaning the time point when mobility event (*METP*) $a_{ij}^{ME}\left(t_{j},l_{j}\right)$ happens is generated by a certain function, $l_{j}=f^{MESL}\left(t\right)⇐p_{ij}\left(t\right)$ meaning which spatial location individual $a_{i}^{ind}$ moves (*MESL*) to at time $t_{j}$ is generated by a certain function defined in Equation (2).

#### 3.1.8. Formulation of Individuals’ Contact Activities

We also represented individual heterogeneous contact patterns and scheduled contact activities as discrete events in queue models. Individual contact activity matrix is formulated as

(30) $$M^{CE}=\left[\begin{array}{ccc} a_{11}^{CE}\left(t_{1},b_{1},d_{1}\right) & \cdots & a_{1k}^{CE}\left(t_{k},b_{k},d_{k}\right)\\ \vdots & \ddots & \vdots \\ a_{n1}^{CE}\left(t_{1},b_{1},d_{1}\right) & \cdots & a_{nk}^{CE}\left(t_{k},b_{k},d_{k}\right) \end{array}\right],$$

where $M^{CE}$ is contact event (*CE*) matrix with *n* rows and *k* columns. $a_{ij}^{CE}\left(t_{j},b_{j},d_{j}\right)$ is the contact event *j* of individual $a_{i}^{Ind}$, which denotes individual $a_{i}^{Ind}$ initiates a contact event with individual $a_{j}^{Ind}$ at time $t_{j}$. Furthermore, the contact activity sustains a duration time $d_{j}$. The constraint conditions $C^{CE}$ of contact event matrix include $i<j\Rightarrow t_{i}<t_{j}$ meaning contact events are scheduled in time sequence, $t_{j}=f^{CETP}\left(t\right)$ meaning the time point (*CETP*) when contact events happen is generated by a certain function, $b_{j}=f^{CEO}\left(t\right)⇐q_{ij}\left(t\right)$ denoting individuals select contact objects (*CEO*) according to a function defined in Equation (6), and $d_{j}=f^{CED}\left(t\right)⇐d\left(r\right)$ meaning the duration time of contact activities (*CED*) is generated by a certain function defined in Equation (4).

### 3.2. Statistical Properties of Epidemic Diffusion

To conduct theoretical analysis of epidemic spreading regularities, we derived analytical results from matrix-based formulation of entities above. The numbers of susceptible, latent, infectious, and recovered individuals at time *t* were formulated as

(31) $$\{\begin{array}{c} s\left(t\right)=\sum_{k}a_{k1\left(S\right)}^{HS}\left(t\right)\\ l\left(t\right)=\sum_{k}a_{k2\left(L\right)}^{HS}\left(t\right)\\ i\left(t\right)=\sum_{k}a_{k3\left(I\right)}^{HS}\left(t\right)\\ r\left(t\right)=\sum_{k}a_{k4\left(R\right)}^{HS}\left(t\right) \end{array},$$

The number of new infections (*NI*) at time *t* is derived as

(32) $$n^{NI}\left(t\right)=\sum_{k}a_{k4\left(R\right)}^{HS}\left(t\right)-\sum_{k}a_{k4\left(R\right)}^{HS}\left(t-1\right)+\sum_{k}a_{k3\left(I\right)}^{HS}\left(t\right)-\sum_{k}a_{k3\left(I\right)}^{HS}\left(t-1\right).$$

The accumulated number of infected individuals (*AI*) is derived as

(33) $$n^{AI}\left(t\right)=N^{Ind}-\sum_{k}a_{k1\left(S\right)}^{HS}\left(t\right),$$

where $n^{AI}\left(t\right)$ is the accumulated number of infections at time *t*. Then, we can get final epidemic size as $n^{AI}\left(t_{end}\right)$.

To analyze the spatial patterns of epidemic diffusion, we derived the distribution of infectious individuals at spatial locations (*SL-I*) as

(34) $$p_{k}^{SL-I}\left(t\right)=\frac{\sum_{j}\left(a_{jk}^{CSL}\left(t\right)a_{j3\left(I\right)}^{HS}\left(t\right)\right)}{\sum_{k}a_{k3\left(I\right)}^{HS}\left(t\right)},$$

where $p_{k}^{SL-I}\left(t\right)$ is the proportion of infectious individuals at spatial location $a_{k}^{SL}$ at time *t*. $a_{jk}^{CSL}\left(t\right)a_{j3\left(I\right)}^{HS}\left(t\right)$ is the label denoting whether individual $a_{j}^{Ind}$ is in infectious state and stays at location $a_{k}^{SL}$ at time *t*. $\sum_{k}a_{k3\left(I\right)}^{HS}\left(t\right)$ is the total number of infectious individuals at time *t*. Furthermore, we derived the density of infectious individuals at spatial locations as

(35) $$d_{k}^{SL-I}\left(t\right)=\frac{\sum_{j}\left(a_{jk}^{CSL}\left(t\right)a_{j3\left(I\right)}^{HS}\left(t\right)\right)}{\sum_{j}a_{jk}^{CSL}\left(t\right)},$$

where $d_{k}^{SL-I}\left(t\right)$ is the density of infectious individuals at spatial location $a_{k}^{SL}$. $\sum_{j}a_{jk}^{CSL}\left(t\right)$ is the number of individuals who are at spatial location $a_{k}^{SL}$ at time *t*. Similarly, we got the distribution of infectious individuals at social organizations (*Org-I*) as

(36) $$p_{k}^{Org-I}\left(t\right)=\frac{\sum_{j}\left(a_{jk}^{Org-Ind}a_{j3\left(I\right)}^{HS}\left(t\right)\right)}{\sum_{k}a_{k3\left(I\right)}^{HS}\left(t\right)},$$

where $p_{k}^{Org-I}\left(t\right)$ is the proportion of infectious individuals at social organization $a_{k}^{Org}$ at time *t*. $a_{jk}^{Org-Ind}a_{j3\left(I\right)}^{HS}\left(t\right)$ is the label denoting whether individual $a_{j}^{Ind}$ is in infectious state and belongs to social organization $a_{k}^{Org}$. The density of infectious individuals at social locations is formulated as

(37) $$d_{k}^{Org-I}\left(t\right)=\frac{\sum_{j}\left(a_{jk}^{Org-Ind}a_{j3\left(I\right)}^{HS}\left(t\right)\right)}{\sum_{j}a_{jk}^{Org-Ind}},$$

where $d_{k}^{Org-I}\left(t\right)$ is the density of infectious individuals at social locations $a_{k}^{Org}$. $\sum_{j}a_{jk}^{Org-Ind}$ is the number of individuals who belong to social organization $a_{k}^{Org}$. We derived the reproduce number of epidemics according to transmission network matrix as

(38) $$\{\begin{array}{c} R=\frac{\sum_{i}\sum_{j}a_{ij}^{TN}}{\sum_{i}n_{i}^{TN}},\sum_{i}n_{i}^{tn}\neq 0\\ n_{i}^{TN}=1,\sum_{j}a_{ij}^{tn}\geq 1\\ n_{i}^{TN}=0,\sum_{j}a_{ij}^{tn}=0 \end{array},$$

where *R* is the reproduce number of epidemics. $\sum_{i}n_{i}^{TN}$ is the number of infected individuals who produce more than one of the second-generation cases.

### 3.3. Algorithms of Computation

**Figure 1:**
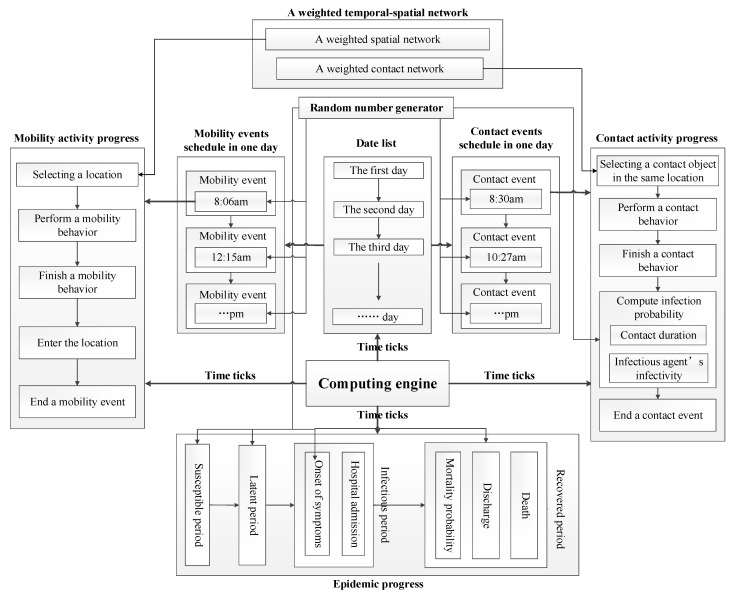
The computational framework of algorithms.

We designed algorithms to force the computation of heterogeneous individual-based epidemic models formulated by matrices and vectors. The computational framework of our models is illustrated in Figure 1.

There are four computational processes invoked in time ticks, which contain eight major modules composed of algorithms in the framework. These modules are described as follows.

1. Computing engine: an engine that forces the computation of models. It advances the computation of models in time order. In each time tick, the engine invokes the computing of mobility activity progress, contact activity progress, and epidemic progress. Furthermore, it realizes time management service that calculates date list. Besides, it invokes events schedules to generate and update mobility events and contact events at the beginning of each day.
2. Mobility events schedule: a container that generates, updates, stores mobility events. At the beginning of each day, it generates new mobility events for each individual to update event schedule.
3. Contact events schedule: a container that generates, updates, store contact events. At the beginning of each day, it generates new contact events for each individual to update event schedule.
4. Mobility activity progress: a progress that executes the computation of a mobility activity including choosing expected location, calculating individual’s position, etc. A weighted spatial network is applied to build the model of individual mobility.
5. Contact activity progress: a progress that executes the computation of a contact activity including choosing expected contact object, calculating contact duration, calculating infectious probability, etc. A weighted contact network is applied to build the model of individual contact.
6. Epidemic progress: a progress that represents the evolution of health states of infected individuals. It fulfills the computation of individual transitions among different health states by using the infected time and the duration of each health state.
7. A weighted temporal-spatial network: a weighted network that combined a spatial network with a contact network. The weighted temporal-spatial network is applied to build the model of individual mobility and contact behaviors.
8. Random number generator: a generator that produces random numbers subject to various distributions. These random numbers are utilized to represent the stochastic nature of epidemic spreading and individual behaviors (e.g., infection probability, mobility time, contact time, contact duration, the durations of health states, etc.).

These modules implement different services in order to execute the computation of epidemic models. It is essential to design algorithms to realize the capabilities of providing these services in these modules. We summarize four major algorithms represented as follow.

#### 3.3.1. Algorithm of Computing Engine

**Figure 2:**
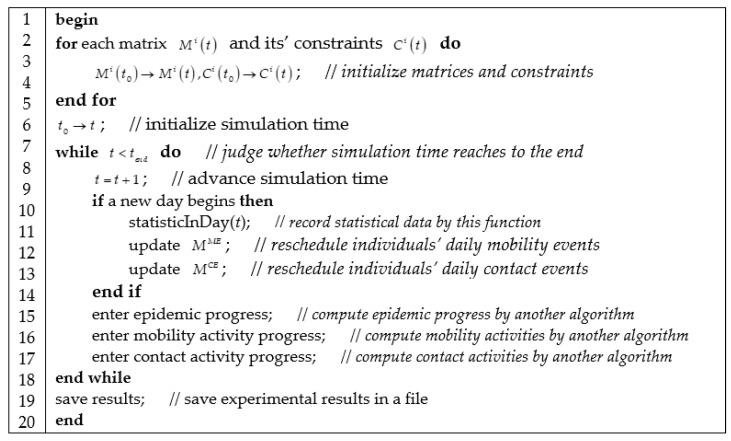
Algorithm of computing engine.

Computing engine implements three jobs described in Figure 2. Computing engine firstly initializes models and parameters described as matrices and constraints. Then, computing engine enters the main loop of calculations and advances the computation of models in time ticks. In each time tick, computing engine invokes epidemic progress, mobility activity progress, and contact activity progress. Meanwhile, computing engine record the state of epidemic diffusion and update mobility and contact event schedules. Computing engine finally outputs analytical results and terminates the computation of models.

#### 3.3.2. Algorithm of Mobility Activity Progress

**Figure 3:**
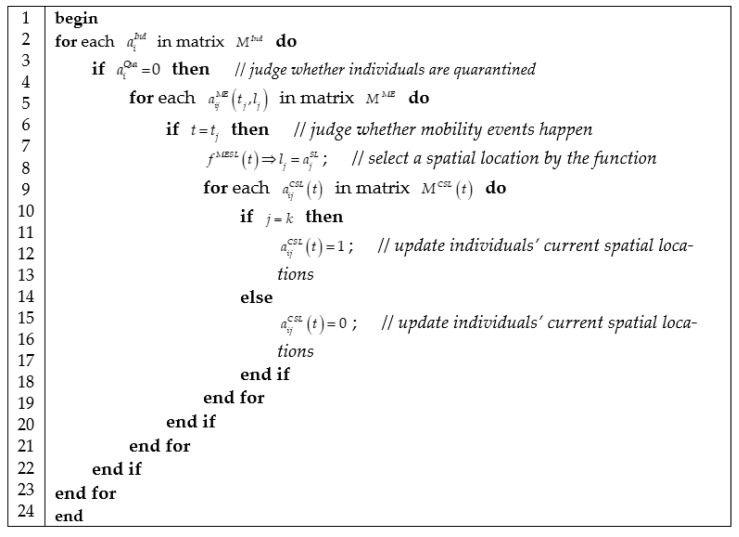
Algorithm of computing mobility activities.

We represent mobility activity progress in Figure 3, which contains three jobs: querying a mobility activity in mobility event schedule; selecting an activity location according to weighted spatial mobility network; and updating the current location matrix.

The function $f^{MESL}\left(t\right)$ in Figure 3 decides which spatial location is selected as individuals’ destination in their daily mobility activities according to the probability formulated as

(39) $$p_{ij}\left(t\right)=\frac{\left(1-a_{j}^{Clo}\left(t\right)\right)a_{ij}^{BN}a_{ij}^{BNW}\left(t\right)}{\sum_{k}\left[\left(1-a_{k}^{Clo}\left(t\right)\right)a_{ik}^{BN}a_{ik}^{BNW}\left(t\right)\right]},$$

where $p_{ij}\left(t\right)$ is the probability that individual $a_{i}^{Ind}$ selects spatial location $a_{j}^{SL}$ as his destination in a daily mobility activity. The probability $p_{ij}\left(t\right)$ is defined by using the proportion of the edge weights of weighted bipartite network. $1-a_{j}^{Clo}\left(t\right)$ denotes spatial location $a_{j}^{SL}$ is not closed in epidemic outbreaks. This equation is a matrix-based formulation of Equation (2).

#### 3.3.3. Algorithm of Contact Activity Progress

**Figure 4:**
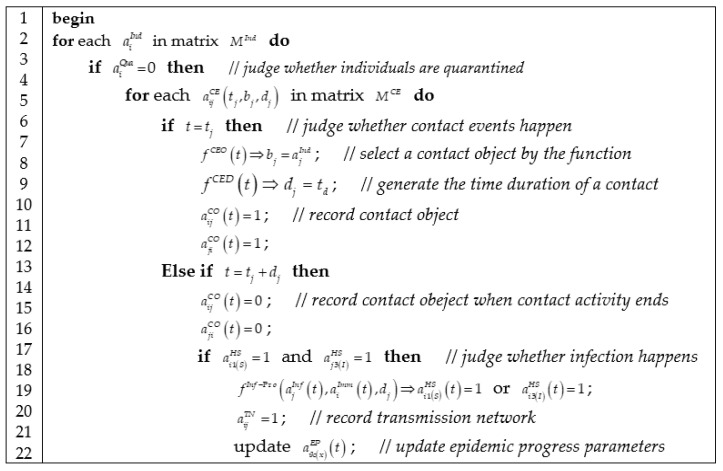
Algorithm of computing contact activities.

We described contact activity process in Figure 4, which contains four jobs: querying a contact activity in contact event schedule; selecting a contact object according to weighted contact network; updating current contact object matrix; and computing the infection at the end of contact activities.

The function $f^{CEO}\left(t\right)$ in Figure 4 decides who are going to contact with whom in individuals’ contact activities according to the probability described as

(40) $$q_{ij}\left(t\right)=\frac{\left(1-a_{j}^{Qua}\left(t\right)\right)\left(\sum_{m}\left[a_{im}^{CSL}\left(t\right)a_{jm}^{CSL}\left(t\right)\right]\right)a_{ij}^{CN}a_{ij}^{CNW}\left(t\right)}{\sum_{k}\left[\left(1-a_{k}^{Qua}\left(t\right)\right)\left(\sum_{m}\left[a_{im}^{CSL}\left(t\right)a_{km}^{CSL}\left(t\right)\right]\right)a_{ik}^{CN}a_{ik}^{CNW}\left(t\right)\right]},$$

where $q_{ij}\left(t\right)$ is the probability that individual $a_{i}^{Ind}$ selects individual $a_{j}^{Ind}$ as a contact object when individual $a_{i}^{Ind}$ initiates a contact event. The probability $q_{ij}\left(t\right)$ is defined by using the proportion of edge weights of weighted contact network. $1-a_{j}^{Qua}\left(t\right)$ denotes individual $a_{j}^{Ind}$ is not quarantined at time *t*. $\sum_{m}\left[a_{im}^{CSL}\left(t\right)a_{jm}^{CSL}\left(t\right)\right]$ ensures individual $a_{i}^{Ind}$ and individual $a_{j}^{Ind}$ are simultaneously at the same spatial location at time *t*. This equation is a matrix-based formulation of Equation (6).

The function $f^{Inf-\mathrm{Pr}o}\left(a_{j}^{Inf}\left(t\right),a_{i}^{Imm}\left(t\right),d_{j}\right)$ decides whether susceptible individual $a_{i}^{Ind}$ is infected by epidemics from infectious individual $a_{j}^{Ind}$ in a contact activity for a time duration $d_{j}$. The infection function is formulated as

(41) $$f^{\inf-pro}\left(t,d_{j}\right)=\{\begin{array}{c} \left(1-e^{-0.019d_{j}}\right)\frac{t-t_{0}}{t^{*}-t_{0}},t_{0}\leq t\leq t^{*}\\ \left(1-e^{-0.019d_{j}}\right)\frac{t_{1}-t}{t_{1}-t^{*}},t^{*}<t\leq t_{1} \end{array},$$

where $t_{0}=a_{j1\left(IT\right)}^{EP}+a_{j2\left(LP\right)}^{EP}$ is the time point when the infectious period of individual $a_{j}^{Ind}$ begins. $t_{1}=a_{j1\left(IT\right)}^{EP}+a_{j2\left(LP\right)}^{EP}+a_{j3\left(IP\right)}^{EP}$ is the time point when the infectious period of individual $a_{j}^{Ind}$ ends. $t^{*}=t_{0}+10$ (days) is the time point when the infectivity of infections individual $a_{j}^{Ind}$ reaches the maximum. This equation is a matrix-based formulation of Equation (10). 

#### 3.3.4. Algorithm of Epidemic Progress

**Figure 5:**
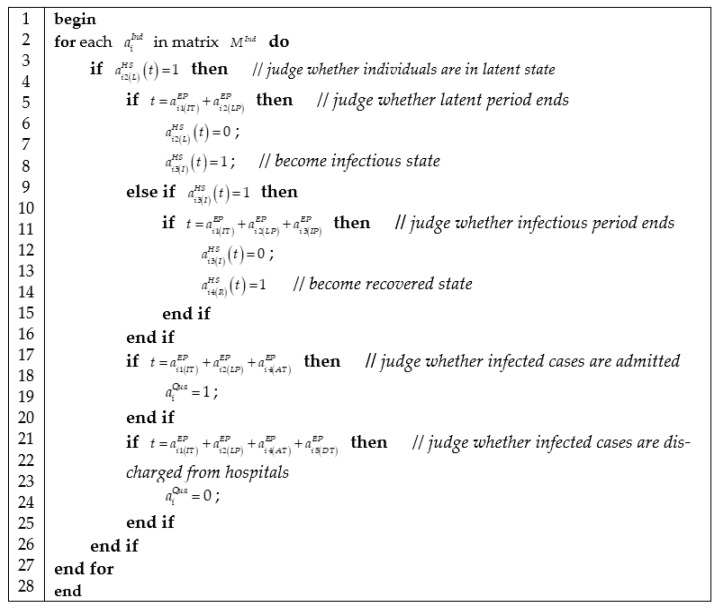
Algorithm of computing epidemic progress.

We represented epidemic progress in Figure 5, which contains three jobs: checking the health state of individuals according to health state matrix $M^{HS}\left(t\right)$; judging whether the current health state of individuals is over according to epidemic progress matrix $M^{EP}$; and then updating health state matrix.

## 4. Simulating the Spread and Control of SARS Epidemic

To test and verify matrix-based formulation of heterogeneous individual-based transmission models of the SARS epidemic, we used the matrix-based models and algorithms to simulate the spread and control of the SARS epidemic.

### 4.1. Experiment Design

We assumed a closed society containing $N^{Ind}=1000$ individuals and $N^{SL}=250$ spatial activity locations. We used the method of randomly adding edges to build a bipartite network and a contact network. The edge weights of bipartite network were generated by a truncated power-law random variable w(r)∈[1,1000]. The exponent ρ of power-law distribution was set as 1.5 in Equation (1) according to refence [31]. The edge weights of contact network were generated according to Equation (5) whose proportional parameter was set as 0.1, and scaling parameter was set as 1.

We assumed each individual executed five mobility activities in a day. Individuals’ daily contact number approximates to a normal distribution with mean 8.6 (standard deviation 4.25) [34]. The time duration of contact activities was represented as a truncated power-law random variable in the range [1,120] minute with an exponent 1.5 [31].

We studied the effect of non-pharmaceutical interventions on the SARS epidemic diffusion, including contact tracing and workplace closure. In case susceptible individuals ever contacted with infectious individuals were found out, they were quarantined. Furthermore, once quarantined individuals had the onset of symptoms, they were admitted to be infected by the SARS epidemic. The admission time of traced infectious individuals was changed to be a Gamma distribution with a mean (standard deviation) of 1 (0.25) day. In case workplaces were closed, individuals stayed at home or had activities in other spatial locations. The changes were realized by revising bipartite network matrix, contact network matrix.

### 4.2. Experimental Results

**Figure 6:**
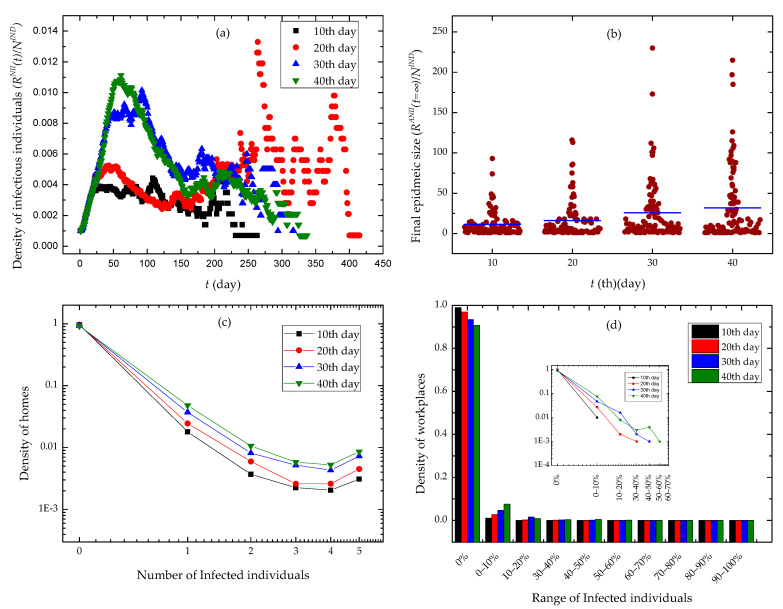
Experimental results. The different days in the legends of subfigures denote the days until workplace closure and contact tracing were implemented. (**a**) The time evolution of the density of infectious individuals. (**b**) Final epidemic sizes with different days until contact tracing and workplace closing policies were implemented. (**c**) The distribution of the density of homes via the number of infected individuals. (**d**) The distribution of the density of workplaces via the range of infected individuals.

We randomly chosen an individual as the infection source of the SARS epidemic, and executed the epidemic control policies of contact tracing and workplace closure at various time in simulation experiments. We conducted a set of 100 experiments for each group of control policies, and illustrated experimental results in Figure 6.

We observed the evolution of the density of infectious individual over time in Figure 6a. The slower we executed a workplace closing policy, the higher peak the curves got. Moreover, the curves quickly drop down after the peak, and may have serious oscillations in the second half of time. In Figure 6b, we found that with the increase of time when we began to use workplace closing policies, the data points denoting the final epidemic size in experiments scattered at the positive direction of Y axis. The final epidemic size has a higher mean value. In Figure 6c,d, we found that the densities of home places and workplaces approximated to power distributions over the number of infected individuals and the range of infected individuals, respectively. Furthermore, in case we adopted a workplace closing policy earlier, the epidemic invaded a fewer families and workplaces.

We found the correlation effect caused by contact activity heterogeneity and activity location heterogeneity in Figure 6. Individuals’ contact events are randomly scheduled in a single day according to a uniform distribution. Moreover, individuals conduct five mobility events in a single day and have activities at different locations in the same moment. When executing a contact event, an individual is only able to contact a neighbor that is also in his current activity location. So, the heterogeneities in the beginning time of contact events, the time duration of contacts, the contact probabilities between neighbors, the beginning time of mobility events, and the probabilities of activity locations bring up temporal-spatial constraints for individuals’ contact behaviors. At the same time, the contact patterns of individuals lead to the temporal-spatial characteristics of epidemic diffusion. Due to workplace closure interventions, the epidemic diffusion across different workplaces is mitigated. It is shown in Figure 6d that workplace closure interventions stop most workplaces from epidemics. However, individuals are likely to stay at home when they cannot go to workplaces. The increase of contact behaviors taking place at home enhances epidemic spread among families. This explains why the tails of curves raise up in Figure 6c.

## 5. Conclusions

Individual heterogeneity is a key factor bridging the link between micro behaviors and macro phenomena. As a bottom-up method, individual-based epidemic models exhibit the capability of representing the heterogeneities in epidemic spreading process. However, the complexity of individual-based models results in the limitation of mathematical formulation.

In this paper, we used matrices to formulate heterogeneous individual-based epidemic models. We adopted matrices and vectors to represent entities and their attributes in epidemic systems, such as individuals, individuals’ health states, the infectivity of infectious individuals, spatial locations, and bipartite networks. Then, we derived analytical results of epidemic diffusion from the matrix-based formulation of entities, and designed algorithms to force the computation of heterogeneous individual-based epidemic models. Finally, we applied the SARS epidemic as a case study to test and verify the heterogeneous individual-based epidemic models in matrix-based formulation. In this case study, we investigated the effect of non-pharmaceutical interventions on the SARS epidemic control, including contact tracing and workplace closure. Experimental results indicate that in case of executing workplace closing interventions early, we can obtain a better effect on mitigating SARS epidemic diffusion. Moreover, we also analyzed the correlation effect caused by contact activity heterogeneity and mobility location heterogeneity, as well as the temporal-spatial patterns of epidemic spread.

Heterogeneous individual-based epidemic models formulated by matrices and vectors are scalable and flexible. We could add more matrices into the models to describe more entities in epidemic systems. Furthermore, we could improve the algorithms or design new algorithms to expand the capability of the models. More analytical results could be formulated from matrix-based representations of entities and their attributes. In addition, heterogeneous individual-based epidemic models formulated by matrices and vectors not only can capture individual heterogeneity, but also have the ability of performing theoretical analysis of epidemic diffusion.

## Data Availability

The data presented in this study are available on request from the corresponding author. The data are not publicly available due to privacy.
